# Promucetin, a new C-type lectin-like protein modulates coagulation by activating platelets via GPIb

**DOI:** 10.1590/1678-9199-JVATITD-2025-0003

**Published:** 2025-07-04

**Authors:** Xiao-Qin Yu, Qi-Yun Zhang, Shu-Ting Zhou, Qing-Yu Lu, Qian-Yun Sun

**Affiliations:** 1 State Key Laboratory of Discovery and Utilization of Functional Components in Traditional Chinese Medicine, Guizhou Medical University, Guiyang, China.; 2 School of Pharmaceutical Sciences, Guizhou Medical University, Guiyang, China.; 3 Natural Products Research Center of Guizhou Province, Guiyang, China.

**Keywords:** C-type lectin-like proteins, Platelet aggregation, Coagulation function, Thrombus, Snake venom

## Abstract

**Background:**

Snake venom C-type lectin-like proteins (also known as snaclecs) have anticoagulation and procoagulation effects by targeting platelet or coagulation factor IX/X, suggesting their potential as candidates for new anticoagulant drugs. Therefore, this study aims to evaluate the antiplatelet and antithrombotic effects of a new snaclec from *Protobothrops mucrosquamatus* venom and its potential as an anticoagulant candidate.

**Methods:**

Promucetin was purified through sequential column chromatography, and its molecular mass was determined by SDS-PAGE. The α- and β-chains of promucetin were identified using liquid chromatography-mass spectrometry (LC-MS). *In vitro* analyses of platelet aggregation were performed using turbidimetric methods, thromboelastography, and coagulation activity assays. For *in vivo* experiments, promucetin was administered to rats at varying concentrations, and platelet changes were monitored. The antithrombotic effects of promucetin were assessed using a FeCl₃-induced rat thrombosis model.

**Results:**

Promucetin existed as two multimers with molecular weights of 140.1 kDa and 91.9 kDa under non-reducing conditions. Sequence analysis revealed that its α-chain and β-chain shared 71% and 34% homology, respectively, with TMVA from the same snake venom. *In vitro* platelet aggregation assays indicated that promucetin activated platelets via glycoprotein Ib. Thromboelastography showed that promucetin inhibited both coagulation factor activity and platelet function, resulting in an anticoagulant effect. Specifically, thrombin time was prolonged, while activated partial thromboplastin time and prothrombin time remained unchanged. *In vivo*, promucetin administration led to a dose-dependent decrease in platelet count. At doses of 25 and 50 μg/kg, promucetin significantly inhibited thrombosis, with inhibition rates of 40.9% and 74.4%, respectively. For comparison, lysine acetylsalicylate produced an inhibition rate of 36.7%.

**Conclusion:**

Promucetin exhibits significant ability to modulate coagulation function and effectively inhibit thrombosis by activating platelet via GPIb and reducing platelet count, which helps us understand its biological function in snake bites, it exhibits the potential to be a candidate for anticoagulant therapy.

## Background

Snake venom C-type lectin-like proteins (CLPs) are a class of proteins characterized by the absence of enzymatic activity [1-3]. C-type lectins have Ca^2+^ dependence and sugar binding ability, while the vast majority of CLPs have lost their sugar binding ability. Based on their ability to bind carbohydrates, they are categorized into two groups: snake venom C-type lectins which exhibit sugar recognition activity dependent on Ca^2+^, and snake venom C-type lectin-like proteins which lack such recognition activity. Because of confusion with classic C-type lectins and since names such as C-type lectin-like or related proteins are frequently abbreviated to CTL or CLP and provide no information about the heterodimeric structure, loop-swapping or higher order multimerization, this group have been named snaclecs (snake venom C-type lectins related proteins, CLRPs) [4]. The fundamental structure of snaclecs comprises two polypeptide chains: the α-chain (14-15 kDa) and the β-chain (13-14 kDa). These chains are bonded together through disulfide bonds to form heterodimers, which can exhibit various oligomeric forms, including αβ, (αβ)_2_, and (αβ)_4_ [5, 6]. The amino acid sequences of snaclecs derived from different snake venoms display a homology of approximately 30%-90%, indicating structural similarities [7]. Snaclecs are associated with a range of biological activities [5], such as anticoagulation and procoagulant effects, with platelets and coagulation factors as their primary targets.

Snaclecs interact with various platelet receptors and coagulation factors through non-covalent protein-protein interactions. Most identified snaclecs bind specifically to platelet receptors such as GPIb, GPVI, α_2_β_1_, and CLEC-2 to either induce or inhibit platelet aggregation. Some snaclecs function as platelet agonists, acting directly on GPIb [8-11] or indirectly via the von Willebrand factor (vWF) [12-15] to induce platelet aggregation. Snaclecs can also activate platelets through one or more receptors [16-19] or potentially unknown targets [20, 21]. TMVA is a platelet agonist that activates platelets via GPIb, it significantly prevents platelet microthrombi formation and prolongs discordant cardiac xenograft survival [22]. In addition, snaclecs can serve as platelet inhibitors, primarily targeting GPIb [11, 23-25] or α_2_β_1_ [26-28]. Anfibatide, a novel GPIb complex antagonist, can inhibit vWF-induced platelet activation and thrombus formation [29, 30]. Beyond their interaction with platelets, snaclecs also influence coagulation factors IX and X, affecting coagulation function and involving anticoagulant proteins such as IX/X-bp [31], IX-bp [32], and X-bp [33]. Bothrojaracin, a snaclec derived from *Bothrops jararaca* snake venom, acts as a potent thrombin inhibitor by binding with high affinity to thrombin exosites [34]. The specificity of snaclecs for these targets suggests their potential as candidates for new anticoagulant drugs or diagnostic tools as well as materials for investigating the structure, function, and evolution of snaclecs.

To date, four snaclecs have been purified from the venom of *Protobothrops mucrosquamatus*: trimecetin, TMVA, mucrocetin, and protocetin [9, 35, 36, 38]. The mechanisms of trimecetin, TMVA, and mucrocetin, acting on platelets are similar, they could directly induce platelets aggregation [9, 35, 36], in which mucrocetin and TMVA induce platelet aggregation via GPIb [9, 35]. Trimecetin did not show affinity to coagulation factors IX and X in the presence of Ca^2+^ ions [37], and no research proves that trimecetin, TMVA, and mucrocetin could act on coagulation factors. Whereas protocetin has dual functions in activating platelet and coagulation factor IX, thereby modulates coagulation *in vivo*, a notable reduction in platelet count and prolonged tail bleeding time [38]. 

The most efficient way for snake venom to reduce platelet function is not by inhibiting the function of individual or several receptors but rather by activating platelets so that they are removed from the circulation producing thrombocytopenia [39]. This study reports the purification of a new snaclec, named promucetin, from the venom of *Protobothrops mucrosquamatus*, detailing its physical and chemical properties, along with its antiplatelet and antithrombotic activities. 

## Methods

### Purification

Lyophilized *Protobothrops mucrosquamatus* venom (PMV) (0.2 g) was dissolved in 1.5 mL of phosphate-buffered saline (PBS). The resulting solution was centrifuged at 2000 rpm/min for 10 min at 4℃. The supernatant was collected and loaded onto a Sephadex G-75 column (2.6 cm × 70 cm) that had been equilibrated with PBS. The column was continuously eluted with PBS at a flow rate of 24 mL/h and samples were collected at 4 mL per tube. Absorbance at 280 nm was measured using a BioTek Synergy multifunction enzyme labeler (Bio-Tek USA, Inc.). Fractions exhibiting platelet aggregation activity were pooled and diluted twofold with 50 mmol/L Tris-HCl (pH 8.9), subsequently applied to a 1 mL HiTrap Q HP column, and further purified using an AKTA Prime protein chromatography system (GE Heathcare, USA). Gradient elution was performed using 50 mmol/L Tris-HCl, 0.5 mol/L NaCl (pH 8.9) at a flow rate of 1 mL/min. Fractions containing components that induced platelet aggregation were pooled, concentrated, and then applied to a Sephacryl S-200 column (2.6 cm × 100 cm) equilibrated with PBS, with elution performed at a flow rate of 20 mL/h. The homogeneity of the samples was assessed by sodium dodecyl sulfate-polyacrylamide gel electrophoresis (SDS-PAGE). The samples were then concentrated and quantified using a BCA kit before being stored at -80°C.

### Electrophoresis

The molecular weight of promucetin was estimated by SDS-PAGE, following the method described by Laemmli [40]. Electrophoresis was carried out on a 12% polyacrylamide gel under both reducing and non-reducing conditions. After electrophoresis, the gel was stained with Coomassie brilliant blue to determine the molecular weight of promucetin.

### Carbohydrate analysis

The bands of promucetin under reducing and non-reducing conditions on SDS-PAGE were stained using periodic acid-Schiff reagent, following the glycoprotein staining methods described by Zhang and Cheng [41]. Photographs were taken and the bands were subsequently stained with Coomassie brilliant blue.

### Mass spectrometry

The bands of promucetin under reducing conditions on SDS-PAGE, specifically the α- and β-chains, were stained with Coomassie brilliant blue. The method followed a previously described experimental protocol, and the α-chain and β-chain of promucetin were analyzed using a liquid chromatograph mass spectrometer (ThermoFisher, USA). 

### Animals

SPF-grade male Sprague Dawley (SD) rats weighing 210^_^260 g were purchased from Sibeifu (Beijing) Biotechnology Co. Ltd [certificate number SCXK (Beijing) 2024-0003], and kept in standard cages with standard laboratory animal feed and water. The rats were housed at a constant temperature of 20-25°C and a relative humidity of 60-70%, with 12 h day/night cycles. Before the experiment, the rats were only allowed free access to water. The experimental animal protocol was approved by the Experimental Animal Ethics Committee of Guizhou Medical University (no. 2402886). All animal experiments and animal welfare experiments were performed in accordance with ARRIVE guidelines.

### Platelet aggregation activity *in vitro*

*Preparation of platelets*

The platelet aggregation assay was conducted using an AG400 platelet aggregation analyzer (Shandong Tailixin Medical Technology Co., Ltd.) at 37℃ using the turbidimetric method [42]. Two SD rats were anesthetized with 40 mg/kg sodium pentobarbital. Whole blood was obtained from the abdominal vein and mixed with 3.2% sodium citrate anticoagulant at a 9:1 ratio. Following centrifugation of the anticoagulated blood at 900 rpm/min for 10 min, platelet-rich plasma (PRP) was collected. The remaining blood was further centrifuged at 3000 rpm/min for 15 min to yield platelet-poor plasma (PPP).

*Preparation of gel-filtered platelets (GFP)*

The main purposes of gel filtration and platelet washing are to obtain platelets without plasma components, for platelet aggregation, results obtained using gel-filtered platelets may be similar to those obtained using washed platelets. However, the mechanical damage caused by washing platelets is greater, so we chose the method of gel filtration to prepare platelets in this study.

Two SD rats were anesthetized using 40 mg/kg sodium pentobarbital, PRP was prepared according to the methods described in previous experiments, and subsequently, the PRP was loaded onto a Sephadex S-400 column that had been equilibrated with Tyrode’s solution. Following the appearance of white turbidity, the solution was collected at a flow rate of 30 mL/h until it became clear. The solution was then centrifuged at 3600 rpm/min for 10 min, the supernatant was discarded, and the remaining precipitate was suspended in Tyrode’s solution to obtain the GFP. 

*Platelet aggregation*

Platelet counts in PRP were adjusted to 3 × 10^8^ platelets/mL using PPP. PRP was pre-incubated at 37°C for 5 min before the addition of promucetin. Specifically, 30 μL promucetin was added to 270 μL PRP, which induced aggregation for 5 min. The final concentrations of promucetin induced PRP aggregation were as follows: 0.2, 0.4, 0.6, 0.8 ng/mL, and 30 μL PBS induced PRP aggregation as the control group, there were three repetitions for each group.

Following the preparation of GFP, platelet counts in GFP were similarly adjusted to 3 × 10^8^ platelets/mL using the Tyrode’s solution. GFP was pre-incubated at 37°C for 5 min before the addition of promucetin, wherein 30 μL promucetin was combined with 270 μL GFP, inducing aggregation for additional 5 min. The final concentrations of promucetin induced GFP aggregation were as follows: 0.07, 0.1, 0.2, 0.8 ng/mL, and 30 μL PBS induced GFP aggregation as the control group, there were three repetitions for each group.

### The effect of cleavage of glycoprotein Ib on platelet aggregation induced by promucetin

Two SD rats were anesthetized with 40 mg/kg sodium pentobarbital, and both PRP and GFP were prepared according to previously described methods. In this context, the platelet counts in GFP were adjusted to 3 × 10^8^ platelets/mL using Tyrode’s solution. Cobra venom metalloproteinase atrase A can cleave the platelet membrane glycoprotein Ib (GPIb), thereby inhibiting platelet aggregation induced by ristocetin and thrombin [42]. In this study, the GFP was treated with atrase A at concentrations of 3.4 and 6.8 mg/mL at 37°C for 5 and 30 min, respectively. Following treatment, 30 μL promucetin (resulting in a final concentration of 0.2 ng/μL) was added to 270 μL GFP to induce platelet aggregation for 5 min.

### Thromboelastography for assays *in vitro*

Three SD rats were anesthetized with 40 mg/kg sodium pentobarbital. Whole blood was collected from the abdominal vein and mixed with a 3.2% sodium citrate solution at a ratio of 9:1. A total of 1 mL of the anticoagulated blood was mixed with 0.1 mL of PBS or promucetin (with a final concentration of 2 μg/mL) and incubated at 37°C for 0, 5, and 10 min. Subsequently, 1 mL of the anticoagulated whole blood was assessed using thromboelastography (Lepu Biotechnology, Ltd, Beijing), following the manufacturer’s instructions.

### Assay for coagulation activity *in vitro*

Two SD rats were anesthetized with 40 mg/kg sodium pentobarbital, and blood samples were anticoagulated using 3.2% sodium citrate (1:9 v/v ratio of citrate to blood). Following anticoagulation, the blood was centrifuged at 3000 rpm/min for 15 min and the supernatant was collected for subsequent experiments. Both promucetin (final concentration: 2 µg/mL) and the supernatant were mixed and incubated at 37°C for 0, 5, and 10 min, respectively, prior to being utilized in the following experiments as plasma samples for detection.

Activated partial thromboplastin time (APTT) was measured by adding plasma (0.1 mL) to the APTT reagent (0.1 mL) that had been pre-incubated at 37°C. The mixture was then incubated at 37°C for 5 min. Afterward, 0.1 mL of 0.025 mol/L CaCl_2_ solution, incubated at 37°C, was added to the mixture and the clotting time was recorded, which was referred to as the APTT value.

Prothrombin time (PT) was assessed by adding 0.1 mL of plasma, incubated at 37°C for 5 min, to 0.2 mL of PT reagent, which had been incubated at 37°C for 3 min, the clotting time was recorded as the PT value.

Thrombin time (TT) was evaluated by diluting the plasma with a diluent for coagulation analysis at a ratio of 1:9. Next, 0.2 mL of plasma, incubated at 37°C for 3 min, was added to 0.2 mL of TT reagent, and the clotting time was recorded as the TT value.

### Effects of promucetin on platelets *in vivo*

Eighty-four SD rats were anesthetized with 40 mg/kg sodium pentobarbital and were intravenously injected with promucetin at doses of 2.5, 5, and 10 µg/kg, respectively. The control group received an equivalent volume of PBS. The number of animals per group was 6 and randomly assigned. Blood samples were collected from the abdominal aorta 0.5, 24, 48, 72, and 120 h post-injection, mixed with EDTA-K_2_ as an anticoagulant, and processed for platelet analysis.

### Antithrombosis assays

Thirty-six SD rats were anesthetized with 40 mg/kg sodium pentobarbital and received intravenous injections of promucetin at doses of 25 and 50 µg/kg, along with 200 mg/kg of lysine acetylsalicylate (LAS). An equivalent volume of PBS was administered to the normal control group rats. The LAS group received treatment 2 h prior to thrombosis induction, whereas the other groups were treated 0.5 h before the same procedure. In antithrombosis assays, there were control, sham, model, promucetin, and positive drug groups, the number of animals per group were 6 and randomly assigned.

The method developed by Kurz et al. [43] was improved to minimize stimulation of the cervical arteries and damage to the vessel surface nerves. Filter papers (1 cm × 0.8 cm) were impregnated with 20% FeCl_3_ solution (20 µL) and were subsequently used to wrap the cervical arteries for 25 min. After this period, the two ends of the thrombi were excised using a blade, and blood samples were collected from the abdominal aorta of the rats. The resulting thrombosis was utilized in subsequent experiments to measure the length, weight, and hematoxylin-eosin staining. Whole blood treated with EDTA-K_2_ anticoagulant was used for platelet assessment, whereas the remaining blood samples, which were anticoagulated with 3.2% sodium citrate (1:9 v/v citrate/blood), were used for thromboelastography. Plasma was utilized to assess APTT, PT and TT following methods described in previous experiments. Fibrinogen (FIB) was measured following the instructions of the assay kits.

### Statistical analysis

Results are expressed as mean ± standard deviation. GraphPad Prism 9.0 software (GraphPad Software, Inc.) was used for statistical analysis and graph plotting. Independent sample t-tests were employed to determine significant differences between two groups, whereas one-way ANOVA was used for comparisons among multiple groups. Statistical significance was set at P ˂ 0.05.

## Results

### Purification

**Figure 1.**
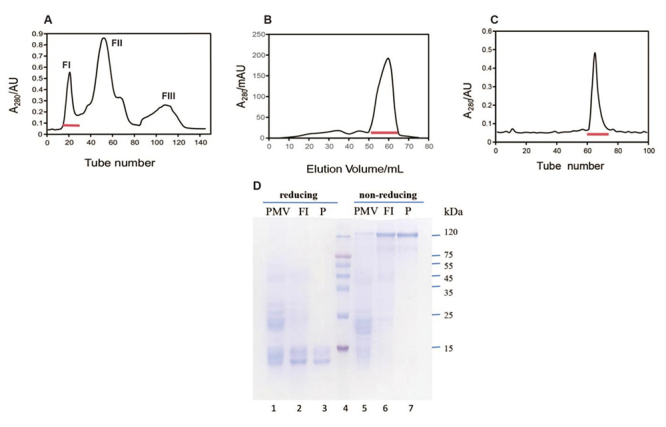
Purification of promucetin. **(A)** Gel filtration of PMV on a Sephadex G-75 column. The protein concentration was estimated from the absorbance at 280 nm after samples were collected at 4 mL per tube. Fractions 13-25 (indicated by the drawing line), that exhibited platelet aggregation activity, were collected and pooled. **(B)** Fractions 13-25 from Sephadex G-75 were pooled, diluted, and loaded onto a 1 mL HiTrap Q HP column. The sample was further purified using an AKTA Prime protein chromatography system. After the samples (indicated by the drawing line) were estimated by SDS-PAGE, the pure and impure samples were collected accordingly. **(C)** The final pure samples (fractions 61-75) from the Sephacryl S-200 column were obtained. **(D)** The homogeneity of promucetin under both reducing and non-reducing conditions was confirmed using SDS-PAGE with a 12% gel. PMV: *Protobothrops mucrosquamatus* venom; FI: the mixture of fractions 13-25 (Figure 1A); P: pure promucetin. Lane 1: PMV under reducing conditions; lane 2: mixture of fractions 13-25 (indicated by the red bar in Figure 1A) under reducing conditions; lane 3: pure promucetin under reducing conditions; lane 4: low-molecular-mass protein standards; lane 5: PMV under non-reducing conditions; lane 6: mixture of fractions 13-25 (indicated by the red bar in Figure 1A) under non-reducing conditions; lane 7: pure promucetin under non-reducing conditions.

Initially, PMV was purified using a Sephadex G-75 column (Figure 1A), resulting in the collection of fractions 13-25 and 30-45, both exhibiting platelet aggregation activity. Subsequently, fractions 13-25 were pooled and further purified using AKTA Prime protein chromatography (Figure 1B). The resulting fractions 54-65 were pooled and concentrated. Finally, the concentrate was loaded onto a Sephacryl S-200 column (Figure 1C), leading to pooling and concentration of fractions 61-75. This preparation was designated as promucetin (> 95% purity). The homogeneity of promucetin was shown by SDS-PAGE under both reducing and non-reducing conditions (Figure 1D).

### Identifying molecular weight through electrophoresis

**Figure 2.**
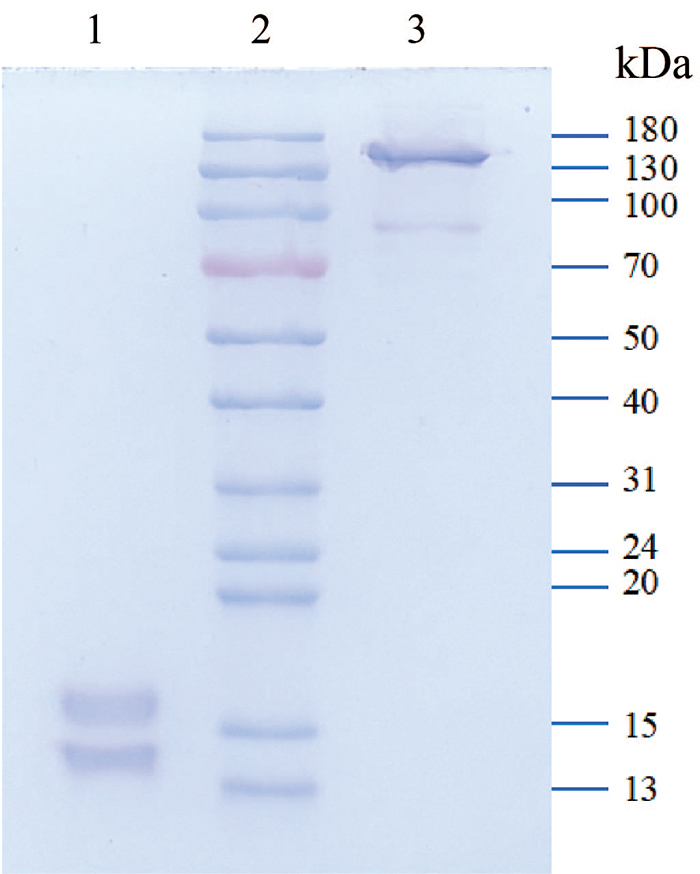
Estimated molecular weight by SDS-PAGE. The electropherogram was dyed with Coomassie brilliant blue. Lane 1: promucetin under reducing conditions; lane 2: low-molecular-mass protein standards; lane 3: promucetin under non-reducing conditions.

The molecular weight of promucetin was estimated by SDS-PAGE under reducing and non-reducing conditions. Under reducing conditions, the molecular weights of the α-chain and β-chain were found to be 15.8 kDa and 14.1 kDa, respectively. Under non-reducing conditions, the molecular weights of the two chains were 140.1 kDa and 91.9 kDa, respectively (Figure 2).

### Glycoprotein staining

**Figure 3.**
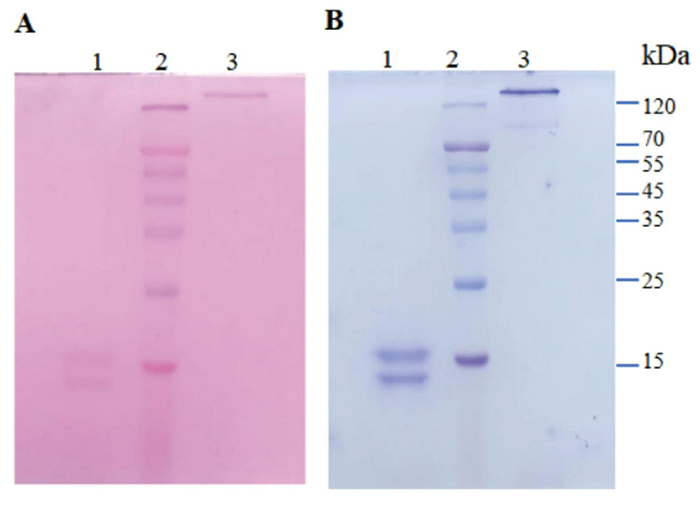
Glycoprotein staining of promucetin. **(A, B)** Both images represent the same electrophoretic pattern, with the only difference being the order of staining. **(A)** The electropherogram was stained with periodic periodic acid-Schiff reagent. Lane 1: promucetin under reducing conditions; lane 2: low-molecular-mass protein standards; lane 3: promucetin under non-reducing conditions. **(B)** The electropherogram was stained with Coomassie brilliant blue.

Based on the results of glycoprotein staining under reducing conditions, the α-chain and β-chain of promucetin were stained with periodic acid-Schiff reagent (Figure 3A, lane 1), and under non-reducing conditions, promucetin also displayed a clear reaction with periodic acid-Schiff reagent (Figure 3A, lane 3). Following imaging, the gel was stained again with Coomassie brilliant blue, which clearly revealed the molecular bands of promucetin under both reducing and non-reducing conditions (Figure 3B). 

### Mass spectrometry

The α- and β-chains of promucetin were separately excised from the electrophoretic gel under reducing conditions and identified using liquid chromatography-mass spectrometry (LC-MS). The homology of the α-chain of promucetin to that of TMVA [10] from the same snake venom was 71%, whereas the homology of the β-chain of promucetin to TMVA [10] was 34% (Table 1).

**Table 1.** Sequence coverage of promucetin and TMVA

Sequence coverage		
α-chain	MGRFTFVSFGLLVVFLSLSGTGADFDCIPGWSAYDR**YCYQAFSEPKNWEDAESFCEEGVKTSHLVSIESSGEGDFVAQLVAEKIKTSFQYVWIGLR**IQNKEQQCR**SEWSDASSVNYENLFKQSSKKCYALKKGTELRTWF NVYCGRENPF VCKYTPEC**	71%
β-chain	MGRFIFVSFGLLVVFISLSGTEAGFCCPLGWSSYDEHCYQVFQQK**MNWEDAEK**FCTQQHTGSHLVSYESSEEVDFVVSK**TLPILKASFVWIGLSNVWNACR**LQWSDGTELMYNAWTAESECIASK**TTDNQWWSMDCSSKR YVVCKF**	34%

Note: matched peptides are shown in bold letters.

### Platelet aggregation activity *in vitro*

*PRP aggregation induced by promucetin*

**Figure 4.**
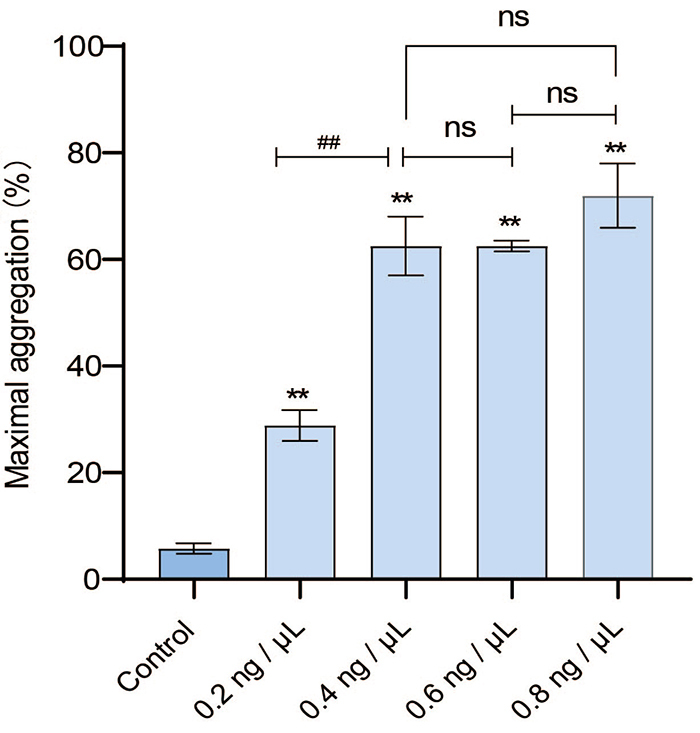
Promucetin induced PRP aggregation in rats. Promucetin effectively induced rat PRP aggregation within 5 min. The results were expressed as the mean ± standard deviation (n = 3), ^**^P < 0.01 compared with the control; ^##^P < 0.01, comparison between administration groups.

Promucetin concentrations were increased from 0.2 to 0.8 ng/μL, resulting in effective induction of PRP aggregation compared to the control group (P < 0.01), with a maximum aggregation rate of 77.03%. Within the concentration range of 0.4 to 0.8 ng/μL, the aggregation rate induced by promucetin exhibited minimal change (P > 0.05, Figure 4). 

*GFP aggregation induced by promucetin*

**Figure 5.**
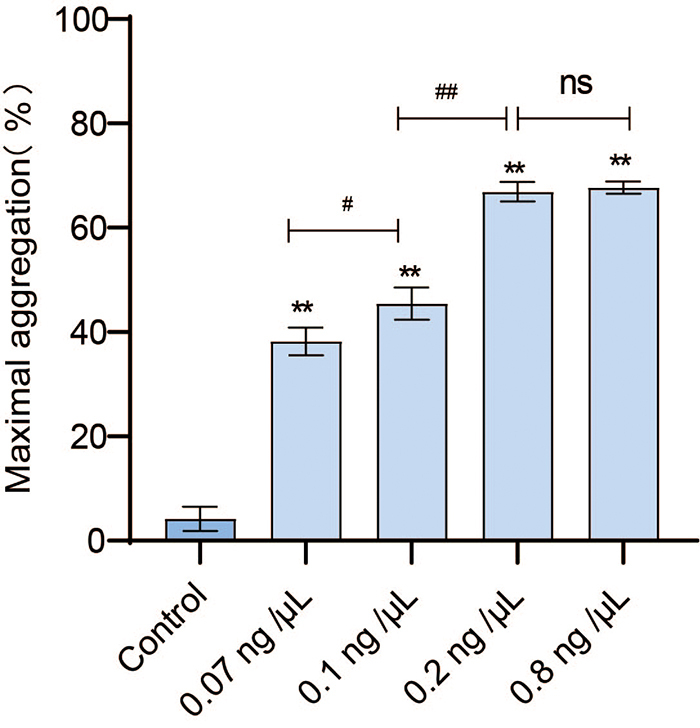
Promucetin induced GFP aggregation in rats. Promucetin effectively induced rat GFP aggregation within 5 min. The results were expressed as the mean ± standard deviation (n = 3), ^**^P < 0.01 compared with the control; ^#^P < 0.05, ^##^P < 0.01, comparison between administration groups.

The concentrations of promucetin were increased from 0.07 to 0.8 ng/μL, demonstrating effective induction of GFP aggregation compared to the control group (P < 0.01). As the concentrations of promucetin were increased from 0.07 to 0.2 ng/μL, the aggregation rate of promucetin on GFP also increased significantly in a dose-dependent manner (P < 0.05, P < 0.01). The aggregation rate changed very little when the concentrations of promucetin were increased from 0.2 to 0.8 ng/μL (P > 0.05, Figure 5).

### The effect of cleavage of GPIb on platelet aggregation induced by promucetin

**Figure 6.**
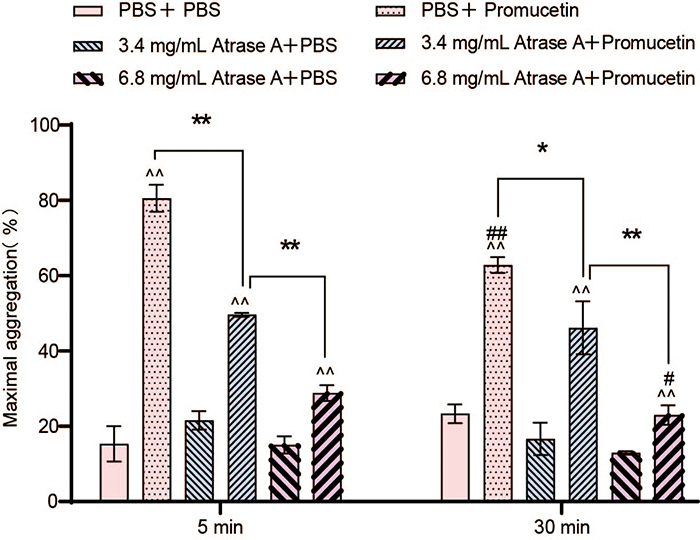
The effect of cleavage of GPIb on platelet aggregation induced by promucetin. The results were expressed as the mean ± standard deviation (n = 3). ^^^^P < 0.01, corresponding control group was compared with corresponding administration group: (PBS+PBS) vs. (PBS + promucetin), (3.4 mg/mL atrase A + PBS) vs. (3.4 mg/mL atrase A + promucetin), (6.8 mg/mL atrase A + PBS) vs. (6.8 mg/mL atrase A + promucetin). ^**^P < 0.01, ^*^P < 0.05, comparison between administration groups: (PBS + promucetin) vs. (3.4 mg/mL atrase A + promucetin), (3.4 mg/mL atrase A + promucetin) vs. (6.8 mg/mL atrase A + promucetin). ^##^P < 0.01, ^#^P < 0.05, comparison between the same administration groups at different times: 5 min (PBS + promucetin) vs. 30 min (PBS + promucetin), 5 min (6.8 mg/mL atrase A + promucetin) vs. 30 min (6.8 mg/mL atrase A + promucetin).

GFP aggregation induced by promucetin was inhibited by atrase A. At final concentrations of 3.4 and 6.8 mg/mL, the aggregation activity of promucetin on GFP was significantly suppressed in a dose-dependent manner (Figure 6).

### Thromboelastography for assays *in vitro*

**Figure 7.**
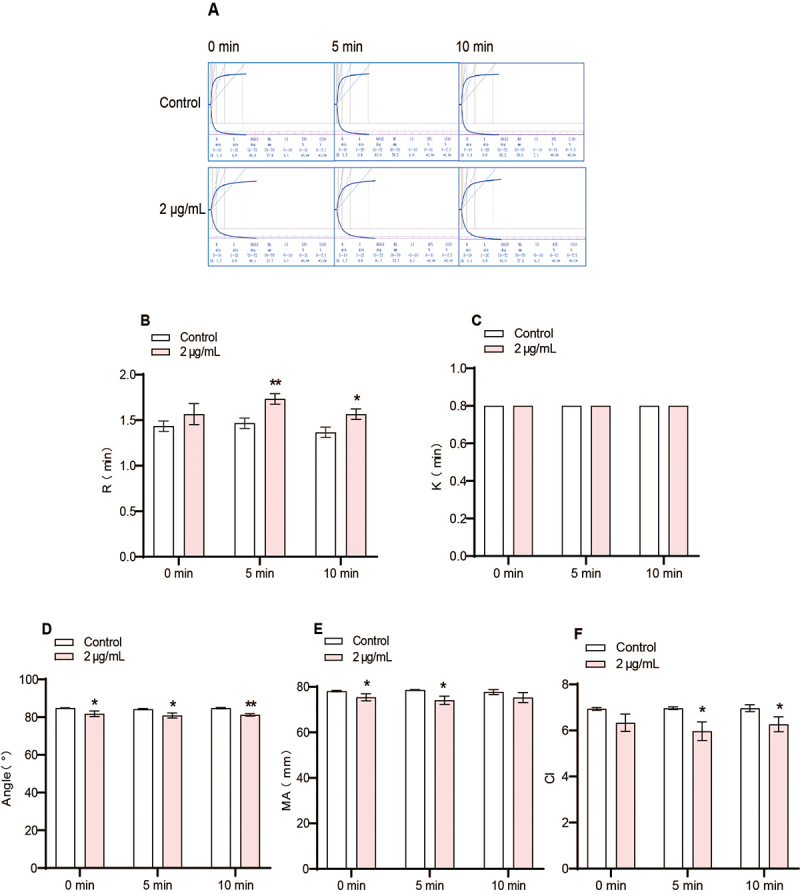
The results of thromboelastography *in vitro*. **(A)** The original data of thromboelastography, **(B)** reaction time (R value); **(C)** kinetic time (K value); **(D)** alpha angle (angle value); **(E)** maximum amplitude (MA value); **(F)** coagulation index (CI value). The results were expressed as the mean ± standard deviation (n = 3), ^*^P < 0.05, ^**^P < 0.01 compared with corresponding control.

Promucetin (at a final concentration of 2 µg/mL) was pre-incubated with anticoagulant blood at 37℃ for periods of 0, 5, and 10 min. The original thromboelastography data are shown (Figure 7A). The results indicate that promucetin had a significant effect on coagulation factors and platelets; however, no obvious time-dependent changes were observed among the three time points. The reaction time (R value), which reflects the overall activity of coagulation factors, was significantly prolonged after pre-incubation for 5 and 10 min (P < 0.05, P < 0.01, Figure 7B). Kinetic time (K value), which indicates the rate of blood clot formation and primarily reflects fibrinogen function, showed no changes after pre-incubation at 0, 5, and 10 min (P > 0.05, Figure 7C). The alpha angle, representing the combined action of fibrinogen and platelets during the initial phase of blood clot formation, significantly decreased across all pre-incubation durations (P < 0.01, P < 0.05, Figure 7D). Maximum amplitude (MA value), indicating the maximum strength of blood clots, mainly influenced by platelets, was significantly reduced after pre-incubation for 0 and 5 min (P < 0.05, Figure 7E). The coagulation index (the CI value), which reflects the overall coagulation status of the sample under these conditions, was markedly lowered after pre-incubation for 5 and 10 min (P < 0.05, Figure 7F). 

### Assay for coagulation activity *in vitro*

**Figure 8.**
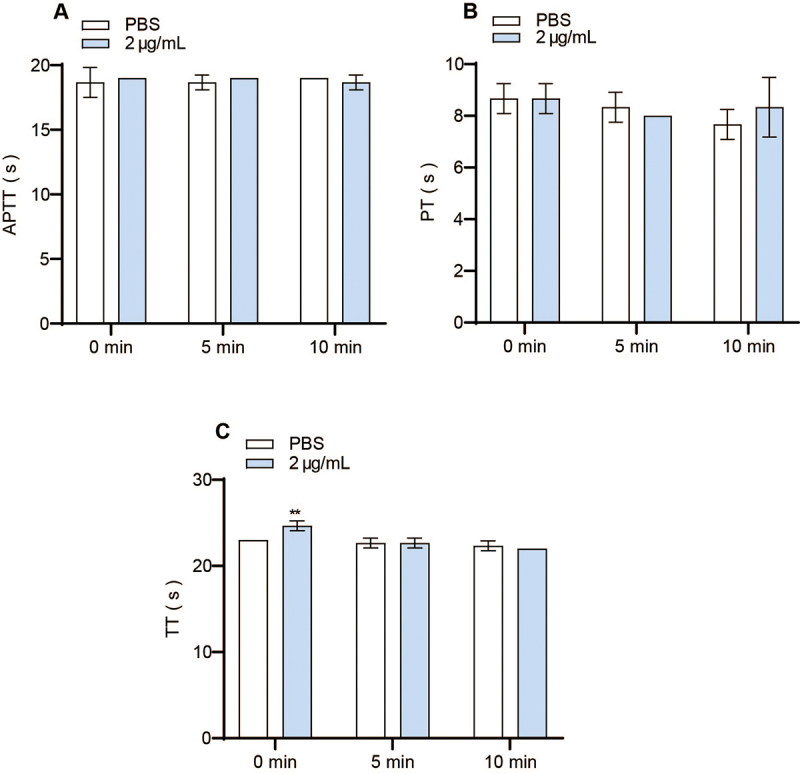
Assay for coagulation activity *in vitro*. **(A)** APTT: activated partial thromboplastin time, **(B)** PT: prothrombin time, **(C)** TT: thrombin time. The results were expressed as the mean ± standard deviation (n = 3), ^**^P < 0.01 compared with corresponding control.

The results indicated that promucetin had minimal impact on APTT and PT *in vitro* (P > 0.05, Figure 8A and 8B); however, TT significantly increased under non-incubation conditions (P < 0.01, Figure 8C).

### Effects of promucetin on platelets *in vivo*

**Figure 9.**
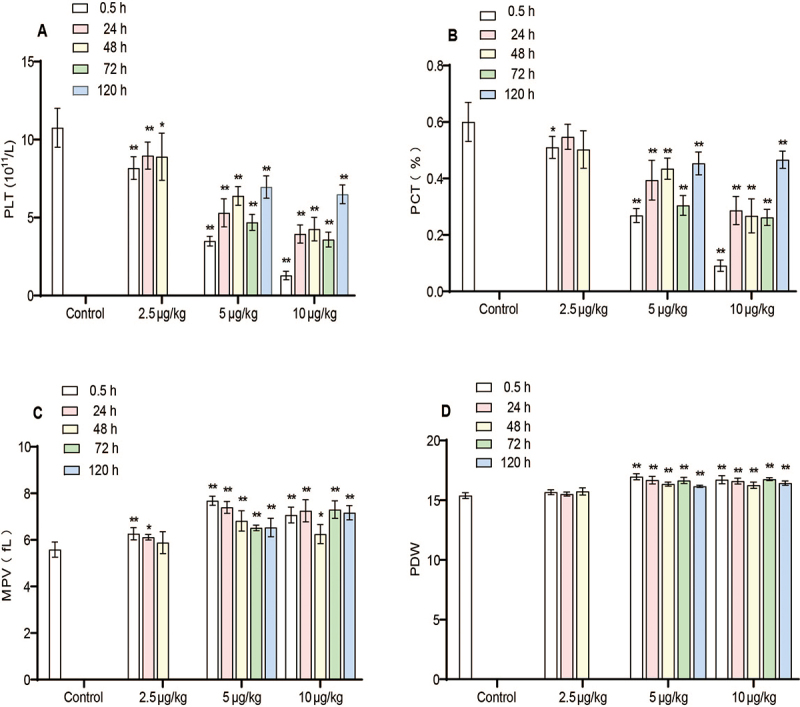
Effects of promucetin on platelets. **(A)** PLT: platelet count; **(B)** PCT: plateletcrit; **(C)** MPV: mean platelet volume; **(D)** PDW: platelet distribution width. The results were expressed as the mean ± standard deviation (n = 6), ^*^P < 0.05, ^**^P < 0.01 compared with control group.

At various time points following administration, the platelet count (PLT) in rats significantly decreased in a dose-dependent manner after the injection of promucetin (P < 0.05, P < 0.01, Figure 9A). The recovery of PLT showed no clear time dependence, and the trend observed in plateletcrit (PCT) mirrored that of PLT (Figure 9A and 9B). The mean platelet volume (MPV) increased after the injection of promucetin (P < 0.05, P < 0.01) and gradually recovered over time, although this recovery did not exhibit a clear time dependence (Figure 9C). Platelet distribution width (PDW) significantly increased in the 5 and 10 μg/kg groups across different administration time points (P < 0.01), whereas the 2.5 μg/kg group showed minimal change (P > 0.05, Figure 9D).

### Antithrombosis assays

*The thrombus weight*

**Figure 10.**
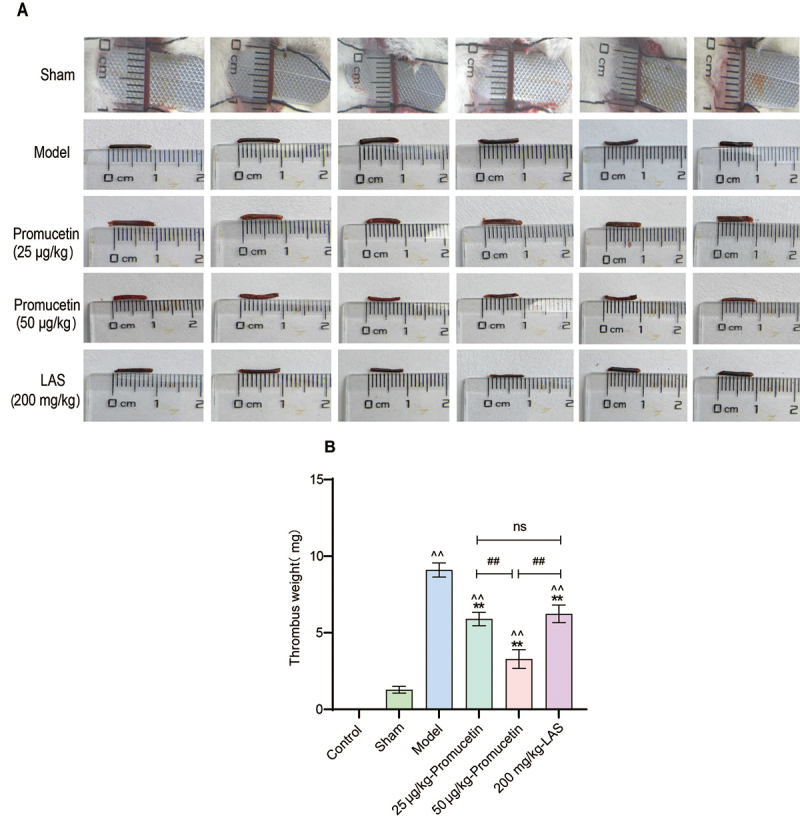
The weight of thrombi. **(A)** Original picture of thrombus length. **(B)** Thrombus weight. The results were expressed as the mean ± standard deviation (n = 6), ^^^^P < 0.01 compared with the sham group, ^**^P < 0.01 compared with the model group, ^##^P < 0.01 compared with 50 μg/kg promucetin group.

The length of all thrombi recorded was 0.9 cm (Figure 10A). The thrombus weight in both the LAS group and the promucetin groups was significantly lower than that in the model group (P < 0.01, Figure 10B). Notably, the thrombus weight in the 50 μg/kg promucetin group was significantly lower than that in the LAS group (P < 0.01, Figure 10B). The thrombosis inhibitory rate for the LAS group was 36.7%, whereas those for the 25 and 50 μg/kg promucetin groups were 40.9% and 74.4%, respectively.

*Hematoxylin-eosin staining*

**Figure 11.**
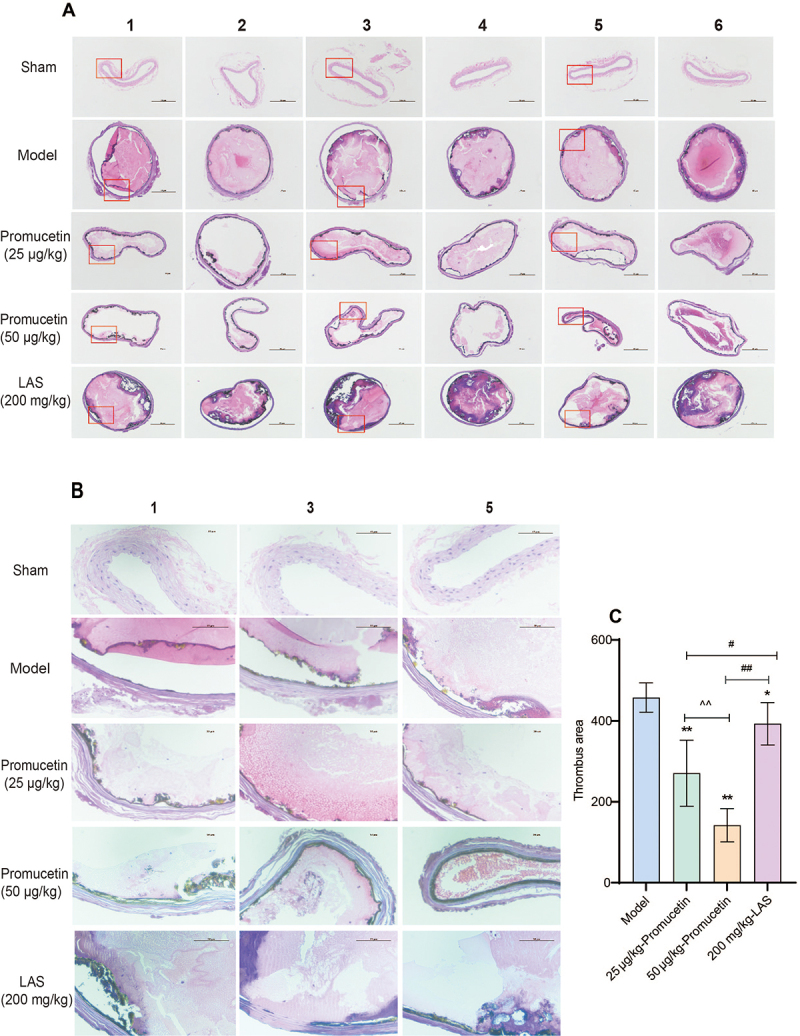
The result of hematoxylin-eosin staining. **(A)** Images captured through a microscope. **(B)** This panel shows higher magnification of the areas in the red boxes in Figure 11A. **(C)** Thrombosis areas in the antithrombosis experiment. The results were expressed as the mean ± standard deviation (n = 6), ^*^P < 0.05, ^**^P < 0.01 compared with the model group; ^##^P < 0.01, ^#^P < 0.05 compared with the LAS group; ^^^^P < 0.01 compared 25 μg/kg promucetin group and 50 μg/kg promucetin group.

The area of arterial thrombosis (Figure 11A) was analyzed using the ImageJ software. Higher magnification images of the sections in red boxes in Figure 11A are displayed in Figure 11B. The thrombosis areas in the promucetin and LAS groups were significantly decreased compared with those in the model group (P < 0.01, P < 0.05, Figure 11C). Furthermore, the thrombosis areas of promucetin groups were significantly smaller than those of the LAS group (P < 0.01, P < 0.05, Figure 11C), whereas the area of the 25 μg/kg promucetin group exhibited a significant decrease compared to the 50 μg/kg promucetin group (P < 0.01, Figure 11C). 

*Thromboelastography for assays in vivo*

**Figure 12.**
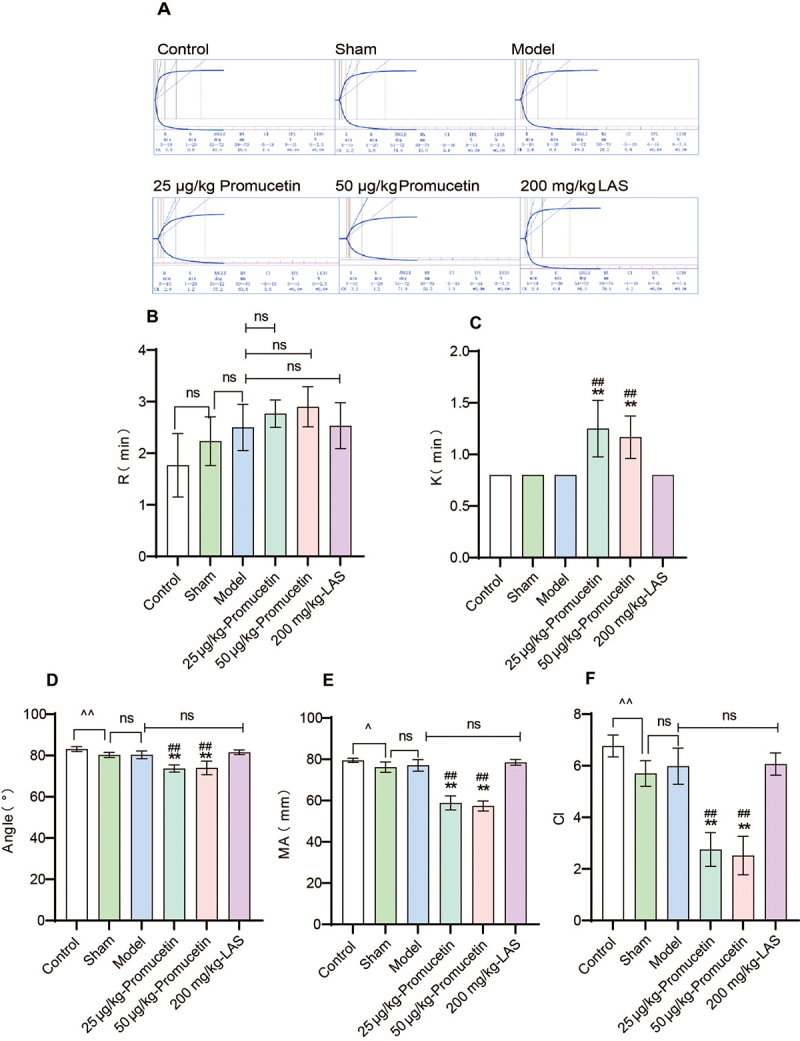
The results of thromboelastography in antithrombotic experiment. **(A)** Original data from thromboelastography; **(B)** reaction time (R value) indicating overall coagulation factor activity; **(C)** kinetic time (K value) representing the rate of blood clot formation, primarily the function of fibrinogen; **(D)** alpha angle (angle value) reflecting the joint action of fibrinogen and platelets during blood clot formation initiation; **(E)** maximum amplitude (MA value) indicating the maximum strength of blood clots, mainly influenced by platelets; **(F)** coagulation index (CI value) representing the comprehensive coagulation status of the sample. The results were expressed as the mean ± standard deviation (n = 6), ^^^P < 0.05, ^^^^P < 0.01 compared with the sham group, ^**^P < 0.01 compared with the model group, ^##^P < 0.01 compared with the LAS group.

The original data from thromboelastography are presented in Figure 12A, revealing that the R value of the promucetin groups (reflecting the overall activity of coagulation factors) showed no significant difference when compared to the model and LAS groups (P > 0.05, Figure 12B). The K value of the promucetin groups (indicating the rate of blood clot formation, primarily determined by fibrinogen) increased significantly (P < 0.01, Figure 12C). Additionally, the angle (reflecting the combined effects of fibrinogen and platelets at the onset of blood clot formation) and MA values (indicating the maximum strength of blood clots) of the promucetin groups decreased significantly (P < 0.01, Figure 12D and 12E). The angle and MA values for the sham group were also significantly lower than those for the control group (P < 0.05, P < 0.01, Figure 12D and 12E). The CI value (indicating the comprehensive coagulation status of the sample) decreased significantly when compared to the model and LAS groups (P < 0.01, Figure 12F).

*Effects of promucetin on platelet in antithrombotic experiment*

**Figure 13.**
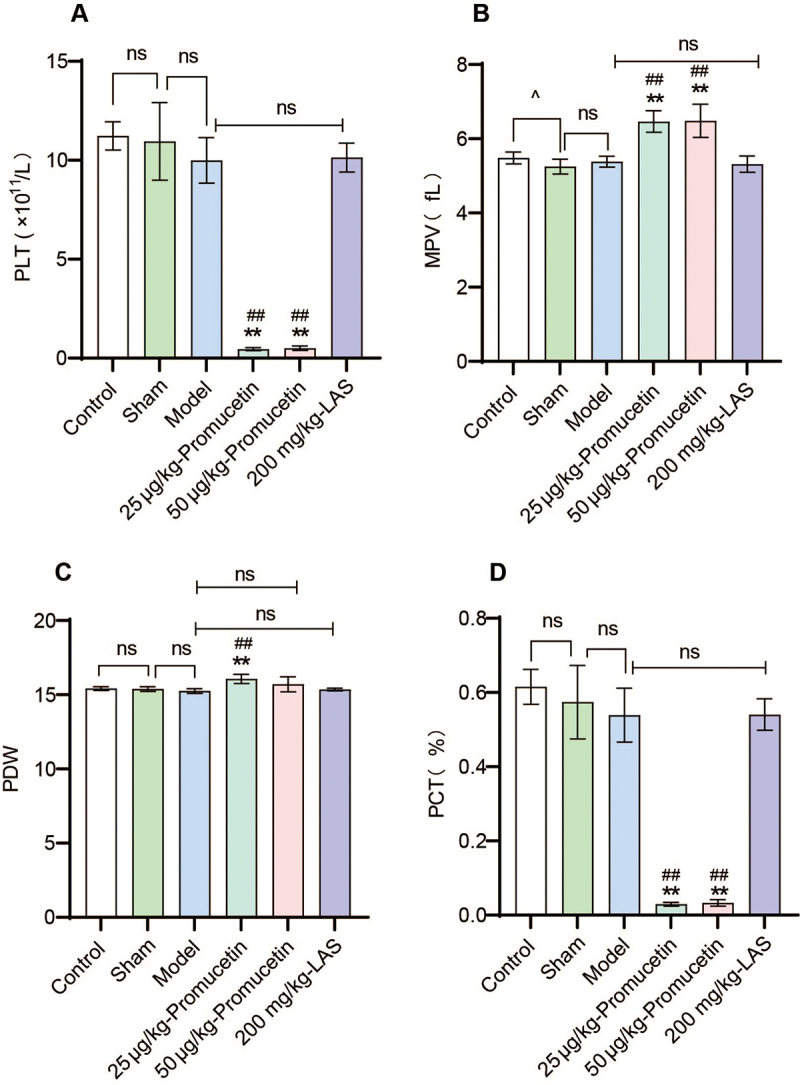
Effects of promucetin on platelet in antithrombotic experiment: **(A)** platelet count (PLT), **(B)** mean platelet volume (MPV), **(C)** distribution width (PDW), **(D)** plateletcrit (PCT), plateletcrit was equal to PLT and MPV. The results were expressed as the mean ± standard deviation (n = 6), ^^^P < 0.05 compared with the sham group, ^**^P < 0.01 compared with the model group, ^##^P < 0.01 compared with the LAS group.

The results indicated that compared with the model and LAS groups, the PLT in the promucetin groups significantly decreased (P < 0.01, Figure 13A), and the MPV in the promucetin groups significantly increased (P < 0.01, Figure 13B). Additionally, MPV in the sham group was significantly lower than that in the control group (P < 0.05, Figure 13B). The PDW in the 25 μg/kg promucetin group showed a significant increase (P < 0.01, Figure 13C), and the PCT in the promucetin groups significantly decreased when compared with the model and LAS groups (P < 0.01, Figure 13D). 

*Assay for coagulation activity in antithrombotic experiment*

**Figure 14.**
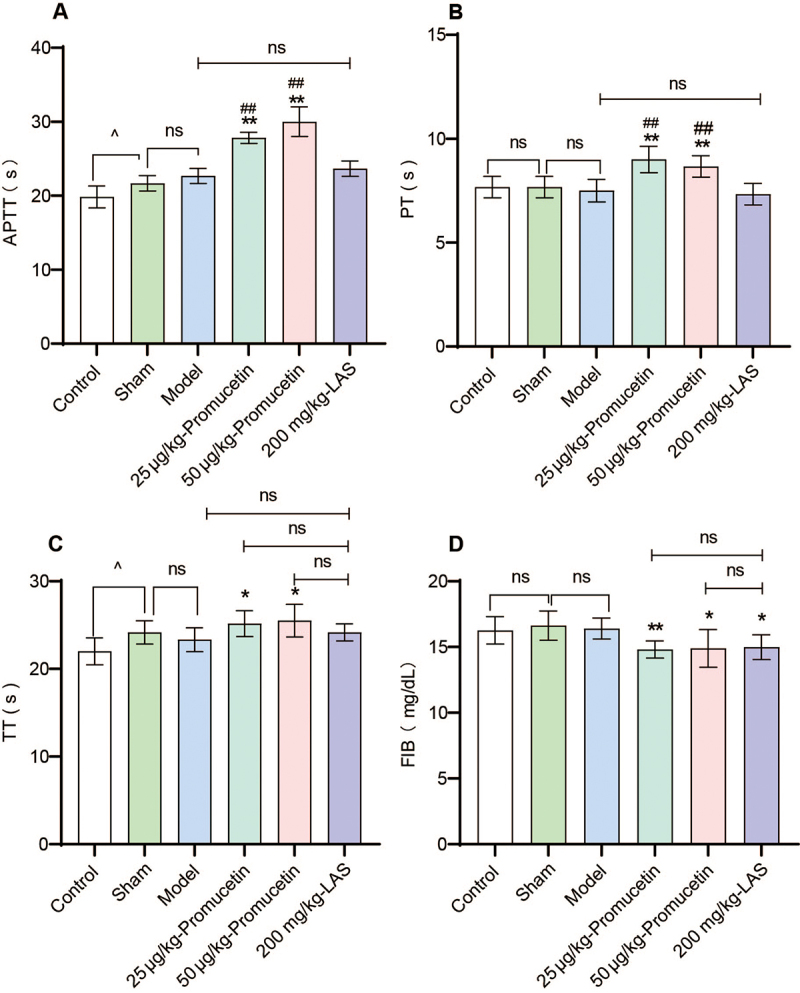
Assay for coagulation activity in antithrombotic experiment. **(A)** APTT: activated partial thromboplastin time, **(B)** PT: prothrombin time, **(C)** TT: thrombin time, **(D)** FIB: fibrinogen. The results were expressed as the mean ± standard deviation (n = 6), ^^^P < 0.05 compared with the sham group; ^*^P < 0.05, ^**^P < 0.01 compared with the model group; ^##^P < 0.01 compared with the LAS group.

In the promucetin groups, APTT, PT, and TT were prolonged (P < 0.05, P < 0.01, Figure 14A, 14B and 14C), whereas the FIB content was significantly reduced compared to that in the model group (P < 0.05, P < 0.01, Figure 14D). Additionally, the APTT and PT of the promucetin groups were significantly extended (P < 0.01, Figure 14A and 14B), although TT and FIB showed no obvious changes when compared with the LAS group (P > 0.05, Figure 14C and 14D). Furthermore, the FIB content in the LAS group was significantly lower than that in the model group (P < 0.05, Figure 14D). The sham group exhibited significantly prolonged APTT and TT compared with the control group (P < 0.05). However, in the model group, APTT, PT, FIB, and TT showed nearly no significant differences compared to the sham group (P > 0.05).

## Discussion

In this study, a new snaclec, promucetin, was purified from the venom of *Protobothrops mucrosquamatus*. SDS-PAGE analysis revealed that promucetin comprises α-chain (15.8 kDa) and β-chain (14.1 kDa), which exist as two convertible multimers with molecular weights of 91.9 kDa and 140.1 kDa under non-reducing conditions. Promucetin induced platelet aggregation and demonstrated a significant anticoagulation effect, effectively inhibiting thrombosis. 

Promucetin was purified from the venom of *Protobothrops mucrosquamatus* using Sephadex G-75, HiTrap Q HP, and Sephacryl S-200 columns. To date, the snaclecs identified from *Protobothrops mucrosquamatus* venom include trimecetin, TMVA, mucrocetin,and protocetin [9, 35, 36, 38]. TMVA exists as two interchangeable multimers: (αβ)_2_ and (αβ)_4_ with molecular weights of 63.7 kDa and 128.5 kDa, respectively [10]. Mucrocetin exists as an (αβ)_4_ polymer with a molecular weight of 135 kDa [9]. The molecular weights of trimecetin [37] and protocetin [38] are 26.1 kDa and 25.4 kDa, respectively. Our study demonstrated that promucetin has two polymers with molecular weights of 91.9 kDa and 140.1 kDa under non-reducing conditions on SDS-PAGE. Under reducing conditions, promucetin showed two distinct bands corresponding to the α-chain and β-chain, with molecular weights of 15.8 kDa and 14.1 kDa, respectively. The electrophoretic performance of promucetin and TMVA was similar. The homology of the α-chain between promucetin and TMVA was 71%, whereas that of the β-chain was 34%. Mass spectrometry analysis indicated that these molecules were different. Despite their differences, snaclecs exhibit similarities in their function and mechanism of action. GPIb plays a direct role in platelet aggregation induced by trimecetin [36], TMVA (mucetin) [35, 44], and mucrocetin [9], whereas protocetin indirectly induces platelet aggregation by binding to von Willebrand factor [38]. At a concentration of 0.2 ng/μL, the aggregation rates of PRP and GFP induced by promucetin were 28.85% and 60.90%, respectively. 

These results indicate that promucetin not only induces PRP aggregation but also directly stimulates platelet aggregation with higher efficiency. Thus far, the primary target of most snaclecs acting on platelets is GPIb [39], hence we hypothesize that promucetin also targets GPIb. In this study, GFP was treated with atrase A to assess the effect of GPIb cleavage on promucetin-induced platelet aggregation. Atrase A, purified from cobra venom, is a snake venom metalloproteinase that cleaves the platelet membrane glycoprotein GPIb, inhibiting aggregation induced by ristocetin and thrombin [42]. Our results showed that promucetin-induced platelet aggregation was significantly inhibited by atrase A indicating that promucetin activates platelet aggregation through GPIb.

It would be intriguing to investigate the effects of promucetin on platelet activation in rats. Following the promucetin injection, there was a significant reduction in platelet count in a dose-dependent manner, and platelet counts gradually recovered over time. Additionally, plateletcrit decreased, and mean platelet volume and platelet distribution width increased, indicating that mature platelets were cleared after activation by promucetin, whereas immature platelets were retained or generated. Exploring the functional implications of promucetin on platelet activity is of great interest. Within the dosage range of 2.5-10 μg/kg, promucetin was non-lethal for rats, and no behavioral changes or detrimental effects were observed in treated rats even after 120 h post-injection, this suggested that the dosage range may be safe. Research has demonstrated that the components of viper venom, particularly snaclecs, may play a crucial role in inducing cerebral infarction [45]. Specifically, two snaclecs, mucetin and stejnulxin from the venoms of *Protobothrops mucrosquamatus* and *Trimeresurus stejnegeri*, respectively, have been identified as potential factors contributing to thrombotic microvascular disease [46]. In our study, when the whole blood of rats was preincubated with promucetin *in vitro*, results from the thromboelastogram indicated that both coagulation factors and platelet function were inhibited. When rat plasma was preincubated with promucetin *in vitro*, followed by testing with APTT, PT, and TT assays, the results showed that APTT and PT remained unaffected, whereas TT was prolonged. This finding suggests that promucetin does not interfere with the intrinsic and extrinsic coagulation pathways, although the reason for the prolonged TT warrants further investigation. Overall, these results clearly indicate that the anticoagulant effect of promucetin is primarily attributed to its action on platelets. Consequently, we further examined the effect of promucetin on the inhibition of thrombosis formation.

Previous antithrombosis studies involving certain snake venoms have shown that bothrojaracin significantly inhibits thrombus formation in rat models of venous thrombosis [27]. TMVA not only significantly prevents the formation of platelet microthrombi but also prolongs discordant cardiac xenograft survival [22]. Cc-Lec inhibites coagulation factors Xa and IXa which leads to anticoagulant and anti-platelet effects and as result prevent thrombus formation related to thrombin generation, it prohibited platelet aggregation induced by ADP, arachidonic acid and fibrinogen suggesting its interaction with their specific receptors namely P2Y1 and/or P2Y12, TPα and GPIIbIIIa respectively [47]. While promucetin may not act directly on the coagulation factor, and it had anticoagulant and antithrombotic effects by activating platelets and reducing platelet count. Building on the anticoagulation mechanism of promucetin, we utilized the FeCl_3_-induced carotid artery thrombosis model in rats to evaluate its inhibitory effects on thrombosis. Following promucetin administration, thrombosis formation was notably reduced, with both high and low dose groups exhibiting significant anti-thrombotic effects - achieving inhibitory rates of 74.4% and 40.9%, respectively. The inhibitory rate of thrombosis in the LAS group reached 36.7%. Furthermore, the thrombosis areas in the promucetin groups significantly decreased compared to the model and LAS groups. This experiment convincingly demonstrates that promucetin effectively inhibits thrombosis, its antithrombotic doses were non-lethal for rats, and no behavioral changes or detrimental effects were observed in treated rats. In addition to wound bleeding caused by surgery, no other bleeding spots were found during the dissection process. But during the modeling process, the bleeding in the 50 μg/kg group was higher than that in the 25 μg/kg group, this suggested that the degree of the platelet reduction increased with increasing concentrations of promucetin, leading to an increased risk of bleeding.

In the antithrombotic experiment, the results demonstrated that promucetin significantly reduced platelet count in rats, decreased the FIB content in plasma, and notably extended APTT, PT, and TT. Concurrently, thromboelastography results indicated that promucetin reduced coagulation factor levels and inhibited platelet function, contributing to its anticoagulant effects. Notably, only the FIB content significantly decreased in the LAS group compared to the model group, whereas other indicators showed minimal changes in the LAS group. The anticoagulant effects observed through these indicators elucidate why promucetin markedly prevents thrombosis and highlight the differences in anticoagulant activity between promucetin and LAS, further explaining promucetin’s superiority over LAS. Based on coagulation function assays, it is noteworthy that significant changes occurred in coagulation factors, fibrinogen, and platelets during the antithrombotic experiment. However, *in vitro* coagulation function tests indicated that promucetin had almost no effect on both the intrinsic and extrinsic coagulation pathways. Consequently, we propose that microthrombi are formed during platelet activation, resulting in a decreased consumption of coagulation factors. Finally, it is crucial to further investigate the safety and potential clinical applications of the antithrombotic and anticoagulant effects of promucetin. 

This research deepens our understanding of the biological role of promucetin in snakebites. Based on its antithrombotic effects and underlying mechanisms, promucetin shows potential as a candidate for anticoagulant therapy. However, further studies are needed to confirm whether it triggers an inflammatory response, and its possible side effects, particularly the risk of bleeding, must be carefully evaluated.

## Conclusions

In this study, promucetin, a new snaclec, was purified from *Protobothrops mucrosquamatus* venom. It demonstrated a significant ability to modulate coagulation and effectively inhibit thrombosis by activating platelets via GPIb and reducing platelet counts. These findings contribute to a better understanding of promucetin’s biological function and raise compelling questions about its potential as a candidate for anticoagulant therapy. Further studies are needed to evaluate its safety and potential side effects as an anticoagulant agent.

## Abbreviations

APTT: activated partial thromboplastin time; CLEC-2: C-type lectin-like receptor 2; FIB: fibrinogen; GPIb: glycoprotein Ib; GPVI: glycoprotein VI; LAS: lysine acetylsalicylate; MPV: mean platelet volume; vWF: von Willebrand factor; PBS: phosphate-buffered saline; PT: prothrombin time; PLT: platelet count; PCT: plateletcrit; PDW: platelet distribution width; TT: thrombin time; SDS-PAGE: sodium dodecyl sulfate-polyacrylamide gel electrophoresis.

## Availability of data and materials

 All data generated or analyzed during this study are included in this article.
